# Identification of key regulators of pancreatic cancer progression through multidimensional systems-level analysis

**DOI:** 10.1186/s13073-016-0282-3

**Published:** 2016-05-03

**Authors:** Deepa Rajamani, Manoj K. Bhasin

**Affiliations:** Department of Medicine, Division of Interdisciplinary Medicine and Biotechnology, Beth Israel Deaconess Medical Center, Harvard Medical School, Boston, MA 02215 USA; BIDMC Genomics, Proteomics, Bioinformatics and Systems Biology Center, Beth Israel Deaconess Medical Center, Research North Building, Room 380E, 99 Brookline Avenue, Boston, MA 02215 USA; BIDMC Center for Vascular Biology Research (CVBR), Beth Israel Deaconess Medical Center, Harvard Medical School, Boston, MA 02215 USA

**Keywords:** Biomarkers, Meta-analysis, *Multi-omics*, PDAC, Systems biology

## Abstract

**Background:**

Pancreatic cancer is an aggressive cancer with dismal prognosis, urgently necessitating better biomarkers to improve therapeutic options and early diagnosis. Traditional approaches of biomarker detection that consider only one aspect of the biological continuum like gene expression alone are limited in their scope and lack robustness in identifying the key regulators of the disease. We have adopted a multidimensional approach involving the cross-talk between the *omics* spaces to identify key regulators of disease progression.

**Methods:**

Multidimensional domain-specific disease signatures were obtained using rank-based meta-analysis of individual *omics* profiles (mRNA, miRNA, DNA methylation) related to pancreatic ductal adenocarcinoma (PDAC). These domain-specific PDAC signatures were integrated to identify genes that were affected across multiple dimensions of *omics* space in PDAC (genes under multiple regulatory controls, GMCs). To further pin down the regulators of PDAC pathophysiology, a systems-level network was generated from knowledge-based interaction information applied to the above identified GMCs. Key regulators were identified from the GMC network based on network statistics and their functional importance was validated using gene set enrichment analysis and survival analysis.

**Results:**

Rank-based meta-analysis identified 5391 genes, 109 miRNAs and 2081 methylation-sites significantly differentially expressed in PDAC (false discovery rate ≤ 0.05). Bimodal integration of meta-analysis signatures revealed 1150 and 715 genes regulated by miRNAs and methylation, respectively. Further analysis identified 189 altered genes that are commonly regulated by miRNA and methylation, hence considered GMCs. Systems-level analysis of the scale-free GMCs network identified eight potential key regulator hubs, namely E2F3, HMGA2, RASA1, IRS1, NUAK1, ACTN1, SKI and DLL1, associated with important pathways driving cancer progression. Survival analysis on individual key regulators revealed that higher expression of IRS1 and DLL1 and lower expression of HMGA2, ACTN1 and SKI were associated with better survival probabilities.

**Conclusions:**

It is evident from the results that our hierarchical systems-level multidimensional analysis approach has been successful in isolating the converging regulatory modules and associated key regulatory molecules that are potential biomarkers for pancreatic cancer progression.

**Electronic supplementary material:**

The online version of this article (doi:10.1186/s13073-016-0282-3) contains supplementary material, which is available to authorized users.

## Background

Cancer is a complex disease that leads to dysregulation of multiple biological processes and pathways at regulatory, transcriptional and translational domains of central dogma. An extensive amount of genomics, proteomics and epigenetics data has been generated to probe the role of molecules from these domains in different pathways and biological processes involved in cancer pathophysiology. Most studies have concentrated on the individual contribution of molecules in disease pathophysiology, ignoring the interactions between *omics* data from different genomics levels. The generation of integrated system-level networks including the molecules dysregulated at regulatory (miRNA, methylation), transcriptional (genes) and translational (proteins, metabolites) *omics* levels helps in generating a complex network dysregulated in the disease. The analysis of this complex network can shed light on critical pathways and key molecules driving disease progression.

Pancreatic cancer is a very aggressive form of cancer with poor diagnosis and dismal prognosis. Of the exocrine pancreatic cancers, 95 % are pancreatic ductal adenocarcinoma (PDAC), with increasing occurrence in the last decades [1]. Patients are mostly diagnosed at an advanced stage, resulting in poor response to therapies including surgical resection, leading to very low (6 %) 5-year survival rates [2]. The probable early clinical symptoms like thromboembolism and new-onset type II diabetes occur much earlier, though the symptoms are not necessarily associated with pancreatic cancer. The early stage progress of the disease could be much slower than previously thought, giving patients with early diagnosis a much better survival probability [3]. To date, the diagnosis is based on clinical signs and pathology even though the early symptoms are vague and non-definitive. This raises an urgent need for the development of reliable diagnostic, prognostic and therapeutic biomarkers. The availability of diagnostic biomarkers from peripheral body fluids will enable routine screening owing to the ease of testing as opposed to the highly invasive methods used currently for diagnosis, while the prognostic markers of early stage PDAC will give patients a better treatment plan and survival chance. In this context, intensive literature mining efforts have been made [4, 5] to identify possible candidate biomarkers in PDAC diagnosis and treatment. These provide a good starting point to collate the genes identified as important players in PDAC that could serve as prognostic or diagnostic biomarkers, but also need extensive filtering and unification with regards to experimental conditions under which each study was performed. For example, studies on prognostic or predictive biomarkers identified from survival analysis are generally done on patients with specific mutations or patients following cancer therapy with specific adjuvant therapy. The result from each study varies with respect to the identified predictive molecules owing to differences in design strategy. Also, predictive signatures obtained from studies have good predictive power for the subset of patients with a similar disease profile as in the study, but fail in the other possible disease profiles, namely, different causal mutations or different adjuvant therapy. This calls for biomarker identification based on biological behavior of genes in PDAC, independent of subpopulation disease profiles. It also calls for a method to filter genes manifesting a causal relationship with disease pathophysiology from the genes being affected by advancement of the disease process.

Another area that needs to be addressed in identification of biomarkers in PDAC is the basic biological processes affected in PDAC. It has been shown that dysregulation of multiple genomic spaces occurs in many cancers and the resultant disease is a culmination of multiple mutations; dysregulated control mechanisms, like regulatory microRNAs and epigenetic control; and dysregulated genes and proteins. The role of small non-coding microRNAs (miRNA) in regulating gene expression in cancer is being extensively studied. It has been shown the oncogenes and tumor suppressor genes are targets of differentially expressed miRNAs in solid tumors [6]. Pedersen et al. studied differential DNA methylation between normal and PDAC samples to identify epigenetic markers [7]. To understand the underlying biological process, these changes have to be taken into consideration as a whole, thus accounting for the biological cross-talk between these different functional components.

With the availability of data from different *omics* studies and large compilations of *multi-omics* data like Gene Expression Omnibus (GEO) [8], the International Cancer Genome Consortium (ICGC) [9] and The Cancer Genome Atlas (TCGA) [10], it is now possible to compare and contrast cancer profiles from normal profiles in multiple *omics* dimensions. The complex nature of interactions leading to the pathophysiology of cancer warrants the use of systems biology-based approaches to understand the cause–effect relationship between the dysregulated molecules identified by traditional supervised analysis methods. Biological interaction networks provide insights into the biology that is fundamental to the disease process. Additionally, biological networks can help to identify key genes that are critical in pathophysiological cancer networks, thus leading to biomarker identification. These key genes (hubs) in the network regulate a plethora of important functional genes and their deletion, in many cases, results in lethality [11]. It has also been illustrated that interactive networks encompassing multiple *omics* domains can provide better insights about key regulatory (KR) molecules than individual dysregulated gene-based analysis, by taking into consideration the gene interactions [12]. In this study, we integrated the information obtained from multiple *omics* dimensions, namely, the transcriptome, regulatory miRNA and the epigenome, to build a systems-level network to identify the key molecules associated with PDAC. Further functional and survival analysis of the identified KR genes clearly depicted their association with pathways linked to cancer progression and survival. Additional file 1: Figure S1 shows a schematic of the methodology used in this study.

## Methods

### Data collection

Raw data on gene expression, miRNA expression and DNA methylation were obtained from public repositories, namely GEO and TCGA, and were normalized using R statistical software and Bioconductor packages in a platform-specific manner. Details about all the published datasets used in this study (accession numbers/references) can be found in Table 1. The meta-analysis of gene expression data was based on four published studies containing PDAC and normal samples. Affymetrix microarray data were preprocessed using the Robust Multichip Average (RMA) method in R statistical platform [13] using the Bioconductor packages. RMA performed background adjustment, quantile normalization and final summarization of oligonucleotides per transcript using the median polish algorithm [14]. Illumina microarray data were processed and quantile normalized using the Lumi package in Bioconductor [15].

**Table 1:** List of pancreatic ductal adenocarcinoma *omics* datasets used in multidimensional analysis

	Dataset	Platform	Samples		Reference
	accession ID		Normal	Pancreatic cancer	(PMID)
mRNA	GSE15471	Affymetrix Human Genome U133 Plus 2.0	36	36	19260470
	GSE28735	Affymetrix Human Gene 1.0 ST	45	45	22363658
	GSE41368	Affymetrix Human Gene 1.0 ST	6	6	24120476
	GSE43797	Illumina HumanHT-12 V4.0 expression bead chip	5	6	24072181
miRNA	GSE24279	Febit human miRBase v11	22	136	NA
	GSE29352	Febit Homo sapiens miRBase 13.0 plus	20	14	NA
	GSE41369	NanoString nCounter Human miRNA assay (v1)	9	9	24120476
	GSE43796	Agilent-031181 Unrestricted_Human_miRNA_V16.0_Microarray	5	6	24072181
Epigenome	GSE49149	Illumina HumanMethylation450 Bead Chip	29	167	24500968
	PAAD (TCGA)	Illumina Infinium Human DNA Methylation 450	6	6	NA
Survival	GSE21501	Agilent-014850 Whole Human Genome Microarray		132	20644708
	GSE32676	Affymetrix Human Genome U133 Plus 2.0 Array	25	7	22261810

*NA* not available, *PMID* PubMed ID

The pancreatic cancer miRNA datasets used in this study are listed in Table 1. Raw data from the Febit platform were normalized using the quantile normalization method after background correction and summarized with replicate median using the linear models for microarray data (limma) Bioconductor package in R. Data from NanoString nCounter were processed using the R package NanoStringNorm [16] using median background correction and quantile normalization. Agilent data were normalized using the AgiMicroRna package from Bioconductor [17, 18] that includes background correction and quantile normalization. The methylation data on pancreatic cancer were obtained from GEO and TCGA (Table 1). The pre-normalized data were median-centered to remove any batch effects.

### Quality control

The quality of the normalized data from Affymetrix gene expression datasets was assessed using the arrayQualityMetrics package from Bioconductor [19]. Individual array quality was assessed using various quality control plots including M-A plots, and homogeneity between arrays was determined using boxplots and density estimate plots. Outliers were identified using heatmaps and dendrograms based on inter-array expression distances (mean absolute distance of the M-value for each pair of arrays). Datasets that did not show any class-based (i.e., normal or PDAC) clustering or had greater than 10 % outliers were not included in the study.

### Unsupervised analysis

Unsupervised analysis was performed to further ascertain the quality of datasets and identify outliers without biological relevance, based on inter-array correlations using principal component analysis (PCA; prcomp module from the stats package in R). PCA projects multivariate data objects onto a lower dimensional space while retaining as much of the original variance as possible [20, 21]. PCA methodology captures the inherent gene expression patterns in the data and identifies the correlation among biologically distinct samples.

### Supervised analysis to identify differentially expressed molecules

We used the limma package [22] from the Bioconductor project to identify differentially expressed molecules (genes, miRNA or methylation) between pancreatic cancer and normal samples. The sample groups were compared by fitting a linear model for each variable (normalized expression values) and applying empirical Bayes smoothing to identify differentially expressed molecules. The molecules with absolute fold change ≥ 2 and multiple test corrected (51) *P*-value < 0.05 were considered significantly differentially expressed in this conventional analysis approach. The list of differentially expressed molecules from each dataset was compared using Venn diagrams created using jvenn [23] (Additional file 2: Figure S2).

### Meta-analysis of data from the epigenome, transcriptome and regulatory miRNA

Meta-analysis was performed to identify the differentially expressed molecules from multiple datasets using a rank-based approach (i.e., RankProd [24, 25]) in the meta-analysis of microarray (MAMA) R package [26]. RankProd is a non-parametric statistical method that uses the ranks of differentially expressed molecules (between conditions compared) to obtain the combined signature from multiple studies. The fold change of genes from individual studies is transformed to rank of genes across studies, and genes that are consistently differentially expressed in multiple studies are ranked highly. The false-positive predictions were restricted to less than 5 % [false discovery rate (FDR) ≤ 0.05], based on 1000 class label-based random permutations. The differentially expressed genes were identified only on the basis of the FDR *P*-value without any fold change restrictions. The rank products method provides a robust way to overcome heterogeneity among datasets and reduces loss of useful data, which is a major problem associated with the traditional methods of finding consensually differentially expressed genes after strict cutoff-based filtering of differentially expressed genes in individual datasets (see previous section).

Similar rank products-based meta-analysis was performed on multiple regulatory miRNA expression datasets and epigenome datasets to obtain PDAC meta-signatures of regulatory miRNAs and differential methylation, respectively. These gene, miRNA and methylation meta-signatures constitute the multidimensional PDAC signature used for further analysis.

### Defining interactions between multiple *omics* dimensions

#### Transcriptome–epigenome interactions

To determine the interactions between the epigenome and transcriptome, the genes and methylations sites were annotated using official HUGO gene symbol (version hg19 of the human genome). The interactions between methylation and gene expression signatures were deduced based on consensus genes found in both gene and methylation meta-signatures. These genes, which show differential expression as well as hypo/hypermethylation, are considered to be the genes regulated by methylation (methylation-regulated genes) irrespective of the directionality of gene expression or methylation. Biplots of gene expression changes with either methylation or miRNA expression changes were generated to extract any patterns between expression profiles (Additional file 3: Figure S3).

### Transcriptome-regulatory miRNA interactions

The miRNA–gene interactions were determined using the RmiR package [27] from Bioconductor. The algorithm performs consensus miRNA and target gene interaction prediction using multiple algorithms (e.g., miRBase and targetScan) to reduce the chance of false-positive results. The miRNA and target predictions were performed using PDAC gene expression and miRNA meta-signatures identified from the analysis described above. The patterns of expression between target genes and miRNA were determined by generating biplots on the basis of fold change of differentially expressed targeted genes and interacting miRNAs. It was not clear if all these interactions were functional in PDAC as the known interactions were obtained based on sequence similarity. To obtain functional interactions in PDAC from among all the predicted interactions identified using RmiR analysis, an additional filter was applied based on the correlation found between gene and miRNA expression data from the same tissue samples in a previous *multi-omics* study [28]. The differentially expressed genes and miRNAs depicting significant correlation at the expression level calculated using the *cor* function in R (R ≥ 0.8 or R ≤ –0.8) were considered the functional gene–miRNA interactions. Further, the genes that were regulated both by methylation and miRNA were considered genes under multiple regulatory controls (GMCs).

### Pathway and functional analyses

To identify biological pathways significantly overrepresented in miRNA-regulated and methylation-regulated genes, pathway enrichment analysis was performed using Ingenuity Pathway Analysis (IPA) (Qiagen) software. The knowledge base of this software consists of functions, pathways and network models derived by systematically exploring the peer-reviewed scientific literature. It calculates the *P*-value using Fisher’s exact test for each pathway and functional category, according to the fit of the user’s data to IPA databases. The *P-*value measures how likely the observed association between a specific pathway/function and the dataset would be if it were due only to random chance. The functional categories and pathways with FDR < 0.05 were considered to be significantly associated.

### Systems biology analysis

The meta-signatures of miRNA, genes and methylation were utilized in generating the network of interacting molecules from multiple *omics* domains using Cytoscape 2.8, an open source platform for biomolecular network visualization [29]. The gene interaction network was created using the Michigan Molecular Interactions (MiMI) plugin for Cytoscape [30]. The network was created based on molecular interactions from multiple biological interaction databases like BIND, BioGRID, CCSB at Harvard, cPath, DIP, GO, HPRD, IntAct, InterPro, KEGG and PubMed [30]. The gene–miRNA interactions were obtained using RmiR analysis as previously described and incorporated in the comprehensive network generation process from multidimensional PDAC meta-signatures. The methylation meta-signature was not included at this step because it was redundant to our eventual goal of creating a network of GMCs. Subsequently, a comprehensive GMC (CGMC) network was extracted from the global multidimensional PDAC meta-signature network, containing GMCs along with their first interactive neighbors. The first neighbors were included to build a cohesive network around the GMCs because the GMCs themselves represent isolated hubs. Network-based pathways enrichment analysis was performed using the GeneMANIA [31] plugin in Cytoscape. Significant pathways associated with subnetworks of selected genes were determined using the FDR-adjusted hypergeometric test-based Q-values reported by GeneMANIA for a query gene-based search for pathways enrichment.

### Identification of key regulatory molecules from the CGMC network

The network topological parameters were calculated using the CentiScaPe Plugin [32] in Cytoscape. Topological analysis was performed on the CGMC network to identify nodes that are critical for network stability. We performed topological analysis using node stress and neighborhood connectivity parameters from CentiScaPe to calculate an average rank (AR) score for each node. Node stress is a node centrality index calculated by measuring the number of shortest paths passing through a node [33]. The neighborhood connectivity of a node *n* is defined as the average connectivity of all neighbors of *n* [34]. The AR score was determined by the average rank of the nodes with respect to these two indices. The nodes with AR score cutoff ≤10 were considered to be KR hubs.

### Partitioning around medoids analysis

Partitioning around medoids (PAM) [35] is a robust clustering method for partitioning based on a dissimilarity matrix. We used PAM implementation from the cluster package in R to partition the CGMC network around the identified KR hubs based on Euclidean distance of CGMC network gene expression matrix.

### Gene set enrichment analysis

Gene set enrichment analysis (GSEA) [36] was performed (using the javaGSEA desktop application) on the CGMC network to determine the importance of the KR subnetworks (gene sets) created using PAM analysis. The GSEA algorithm determines whether a gene set is specifically enriched at the leading edges of a reference gene list sorted with respect to specified parameter of interest (class-based t-test statistic). The edges of the ranked list contain the most discriminatory genes in the gene list and the gene sets depicting overlap with these edges are significant. The significance of gene set enrichment was determined on the basis of 1000 class-based permutation tests [37]. Gene sets with multiple test-corrected *P*-values less than 25 % (FDR ≤ 0.25) were considered significant.

### Self organizing map clustering and survival analysis of key regulatory genes

To identify group-dependent patterns from the expression profiles of KR hubs identified using systems-level approaches, the self organizing map (SOM) clustering technique was adopted [38]. SOM allows the grouping of gene expression patterns into an imposed structure in which adjacent clusters are related, thereby identifying sets of samples that follow certain expression patterns across KR hubs. We performed sample-based SOM clustering (som package in R) using Pearson correlation coefficient-based distance metrics to form two sample clusters with very distinct expression profiles of KRs. Survival analysis was performed on these clusters using the Kaplan–Meier analysis from the survival package in R [39]. Survival analysis was also performed on individual KRs. The Kaplan–Meier estimate is a non-parametric maximum likelihood estimate of the survival function created based on the number of survivors and non-survivors at any given time point. The results of the survival analysis were visualized using a Kaplan–Meier survival curve. The significance of difference in survival among different groups was estimated using log-rank testing. The results were considered significant if the *P*-values from the log rank test were less than 0.075.

## Results

### Meta-analysis of the epigenome, transcriptome and regulatory miRNA in pancreatic ductal adenocarcinoma

Pancreatic cancer data from multiple biological domains (*omics* data types) were integrated to generate a multidimensional PDAC signature associated with disease progression. The study included 177 gene expression profiles, 224 miRNA expression profiles and 210 DNA methylation profiles from different pancreatic cancer studies. Details about the datasets from which these profiles were obtained can be found in Table 1. After normalization and preprocessing, the individual datasets were analyzed using unsupervised and supervised analysis methods.

#### Gene expression meta-signature associated with pancreatic ductal adenocarcinoma

**Fig. 1:**
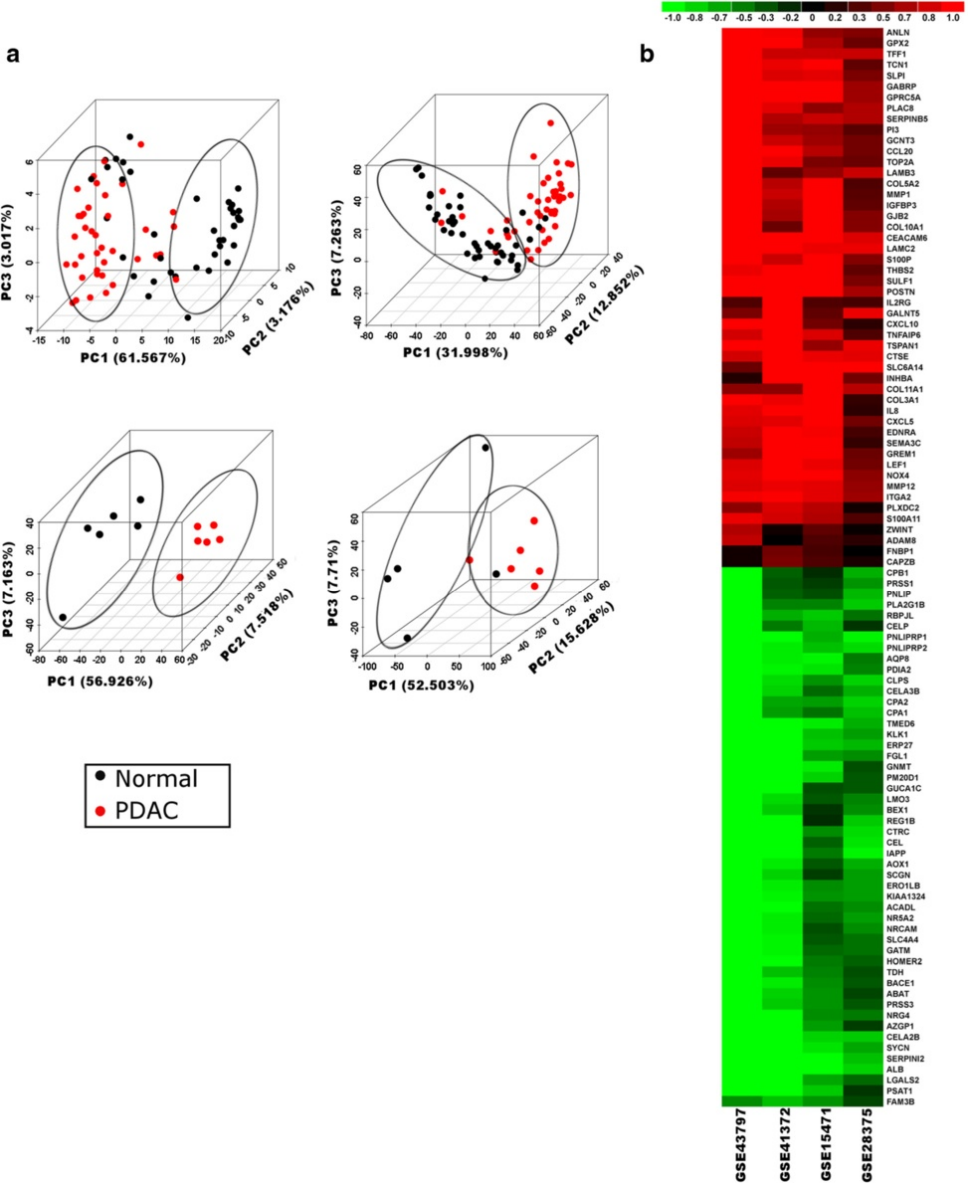
Meta-analysis of pancreatic ductal adenocarcinoma (*PDAC*) transcriptome data. **a** Unsupervised principal component analysis plots across the first three principle components (*PC*) on individual datasets included in the meta-analysis. The variance captured by each PC is shown along the respective axis. PDAC samples are represented in *red* and normal samples in *black*. In all datasets, PDAC and normal samples formed separate clusters marked with ellipses. **b** Heatmap of fold change (*Fc*) of top 100 significantly differentially expressed genes identified from meta-analysis (abs(Fc) ≥ 2 in at least one of the datasets; false discovery rate ≤ 0.05). Datasets are shown as *columns* and genes are shown as *rows*. Relative fold changes are shown with a pseudocolor scale (–1 to 1), with *red* denoting upregulation and *green* denoting downregulation

The unsupervised analysis from transcriptome data using PCA showed good separation between normal and cancer samples (Fig. 1a). In all datasets, normal and PDAC samples were separated with the highest variation (32–61 %) along the first principle component (PC1). Supervised analysis was performed on each dataset to identify differentially expressed genes between normal and PDAC samples using limma. The analysis identified differentially expressed genes in individual gene expression datasets that achieved a FDR ≤ 0.05, and an absolute fold change between normal and PDAC ≥ 2. This individual dataset-based differential expression analysis yielded gene lists with non-significant/small overlap (106 genes out of 5421 total genes) among the datasets (Additional file 2: Figure S2A), indicating heterogeneity among datasets.

Rank-based meta-analysis using the rank products (RP) method was performed to address this inherent heterogeneity among datasets. RP performs a rank-based comparison of genes from different experiments to identify genes that are consistently ranked high (upregulated) and low (downregulated), clearly depicting advantages over linear modeling-based methods in meta-analysis by better handling noise from heterogeneous datasets. The RP-based method depicts a slightly better overlap among differentially expressed genes identified in meta-analysis (Additional file 2: Figure S2A). Owing to this better overlap and the inherent advantage of RP in handling noise, we adopted RP as opposed to limma for meta-analysis. We obtained a transcriptome-specific signature of 5391 genes that were consistently significantly differentially expressed (FDR ≤ 0.05) in PDAC compared to normal among all the datasets. A subset of top differentially expressed genes obtained from meta-analysis is shown in Fig. 1b. It is evident from the heatmap that differentially expressed genes identified from the meta-analysis are consistently over-expressed or under-expressed across all datasets. Many of these differentially expressed genes were initially unidentified because of filtering criteria, but selection using rank-based meta-analysis retained them appropriately. The most downregulated genes identified included genes involved in basic metabolism and functioning of pancreatic cells, for example, *SYCN* involved in exocytosis in pancreatic acinar cells, *FAM3B* involved in controlling basal insulin secretion in pancreatic beta cells, and many enzymes involved in protein processing and amino acid metabolism. The consistently upregulated genes include genes linked to cell cycle progression (e.g., *S100A11*), cytoskeletal reorganization (e.g., *FNBP1* and *CAPZB*), kinetochore formation and spindle checkpoint (e.g., *ZWINT*), immune and inflammatory response (e.g., *IL2RG*, *IL8* and *CXCL5*), angiogenesis (e.g., *PLXDC2*) and metalloproteinase (e.g., *ADAM8*). It was previously shown in a meta-analysis of PDAC expression data that many of these genes are indeed differentially expressed in PDAC [40]. Of the 51 genes shown in that study as potential PDAC biomarkers, 38 were also enriched in our meta-analysis results, including *S100A11*, *ADAM8* and *IL8*.

#### miRNA meta-signature associated with pancreatic ductal adenocarcinoma

**Fig. 2:**
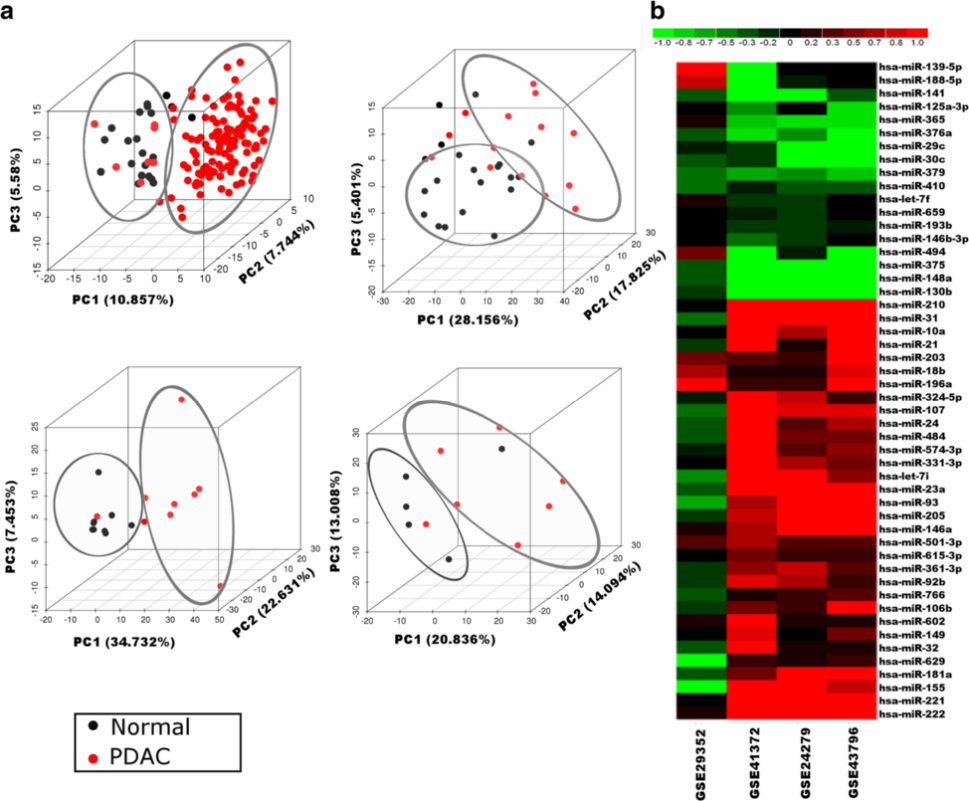
Meta-analysis of pancreatic ductal adenocarcinoma (*PDAC*) regulatory miRNA data. **a** Unsupervised principal component analysis plots across the first three principle components (*PC*) on individual miRNA datasets included in the meta-analysis. The variance captured by each PC is shown along the respective axis. PDAC samples are represented in *red* and normal samples in *black*. In all datasets, PDAC and normal samples formed separate clusters marked in ellipses. **b** Heatmap fold change (*Fc*) of top 50 significantly differentially expressed miRNAs identified from meta-analysis (abs(Fc) ≥ 2 in at least one of the datasets; false discovery rate ≤ 0.05). Datasets are shown as *columns* and miRNAs as *rows*. Relative fold changes are shown with a pseudocolor scale (–1 to 1), with *red* denoting upregulation in PDAC and *green* denoting downregulation

Supervised and unsupervised analyses were performed on normalized miRNA datasets. PCA results showed good separation of PDAC and normal samples along the first three PCs in all datasets (Fig. 2a). PC1 accounted for 10.8–34.7 % of the variance and depicted a significant separation of PDAC and normal samples. As with gene expression data, individual dataset-based differential expression analysis yielded very few miRNAs that were common among the datasets (Additional file 2: Figure S2B), necessitating rank-based meta-analysis.

The miRNA meta-analysis identified 109 consistently significantly differentially expressed miRNAs in PDAC compared to normal, with FDR ≤ 0.05 (miRNA PDAC meta-signature). A heatmap of the top differentially expressed miRNAs obtained from meta-analysis is shown in Fig. 2b. The heatmap clearly depicts uniform upregulation or downregulation of different miRNAs across 3 out of 4 datasets. MiRNAs *miR-148a *and *miR-375 *were significantly downregulated, whereas *miR-93*, *miR-21*, *miR-10a*, *miR-107* and *miR-23a* were significantly upregulated in PDAC. Sun et al. found a similar dysregulation of *miR-148a, miR-375, miR-21 *and *miR-10a *in PDAC [41]. It was shown that *miR-10a* could be involved in the tumor invasiveness through *HOXA1* suppression in PDAC [42]. Our results are consistent with previous findings based on GEO and TCGA data, that *miR-21* is a potential biomarker in pancreatic cancer [43]. *miR-107, miR-222 *and *miR-148a *are differentially expressed in pancreatic cancer and *miR-222 *is also known to be associated with poor survival probabilities [43].

#### Epigenetics meta-signature associated with pancreatic ductal adenocarcinoma

**Fig. 3:**
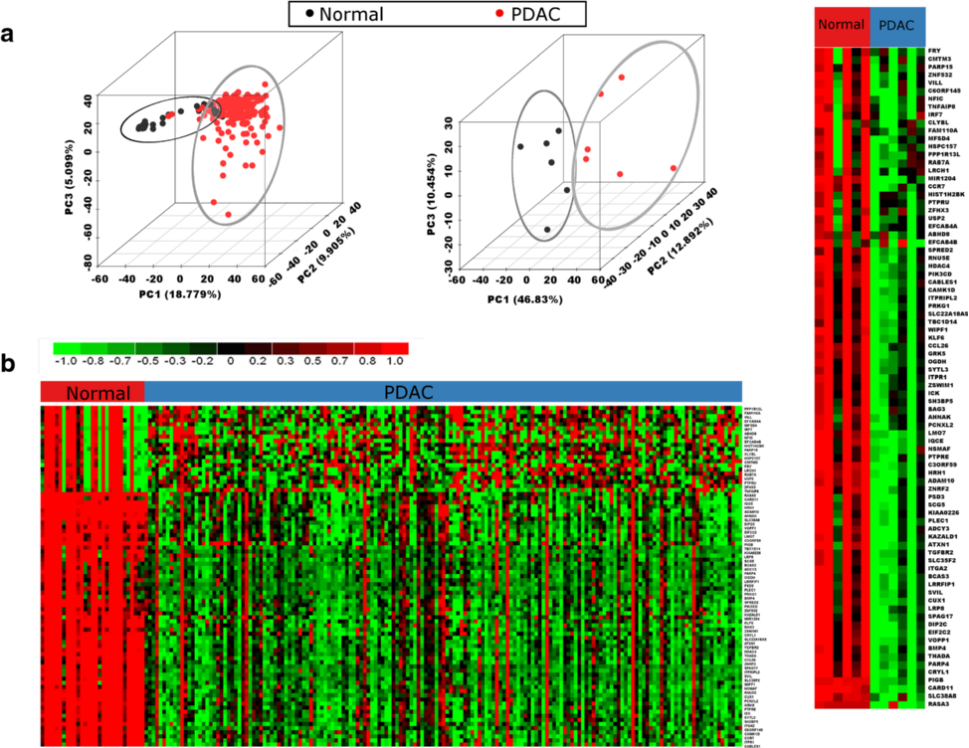
Meta-analysis of pancreatic ductal adenocarcinoma (*PDAC*) epigenome data. **a** Unsupervised principal component analysis plots across the first three principle components (*PC*) on individual DNA methylation datasets included in the meta-analysis. The variance captured by each PC is shown along the respective axis. PDAC samples are represented in *red* and normal samples in *black*. In all datasets, PDAC and normal samples formed separate clusters marked in ellipses. **b** DNA methylation heatmaps show the expression matrix of genes with mean methylation ≥ 0.5 and standard deviation ≥ 0.2 among the top 100 differentially methylated genes identified by rank products analysis (false discovery rate ≤ 0.05) from each dataset used in the meta-analysis. Samples are shown as *columns* and genes as *rows*. Relative methylation is shown with a pseudocolor scale (–1 to 1), with *red* denoting relatively high expression in PDAC and *green* relatively low expression across row-scaled data

Unsupervised analysis of normalized DNA methylation data depicted a significant separation between PDAC and normal samples along the top three PCs in PCA (Fig. 3a). Out of three components, PC1 alone accounted for 18.7 % and 46.8 % variance between PDAC and normal samples in the two methylation datasets. The methylation signature in PDAC constituted 2081 genes that were significantly hyper-methylated or hypo-methylated in PDAC compared to normal (FDR < 0.05). The heatmaps of gene expression values for significantly differentially methylated genes with mean methylation ≥ 0.5 and standard deviation ≥ 0.2 among the top 100 genes identified from RP analysis are shown in Fig. 3b. The top hypo-methylated genes included *CUX1*, a member of the homeodomain family of DNA binding proteins, with a probable role in cell proliferation and cell cycle progression; *CABLES1*, involved in cell cycle regulation through interactions with cyclin-dependent kinases [44]; *RASA3*, a Ras GTPase activator involved in the control of cellular proliferation; *WIPF1,* involved in cytoskeletal organization; *STK17B*, a serine/threonine kinase acting as a positive regulator of apoptosis; and *TGFBR2*, involved in multiple processes related to cell proliferation, differentiation, immunity and carcinogenesis in the cell. Many genes involved in specialized cellular functions were found to be hyper-methylated, for example, *PRKCB*, (protein kinase C), which plays an important role in B-cell activation; *HOXD6* belonging to the homeobox family of genes; *NTM*, a neural cell adhesion molecule; and *IGF2BP1* (insulin-like growth factor 2), an mRNA binding protein involved in translational regulation.

The multidimensional PDAC signature consists of all significantly differentially expressed genes, miRNAs and methylation sites in PDAC identified from the meta-analyses (FDR ≤ 0.05) as described above.

### Understanding gene–miRNA and gene–epigenetic bimodal interactions associated with pancreatic ductal adenocarcinoma

**Fig. 4:**
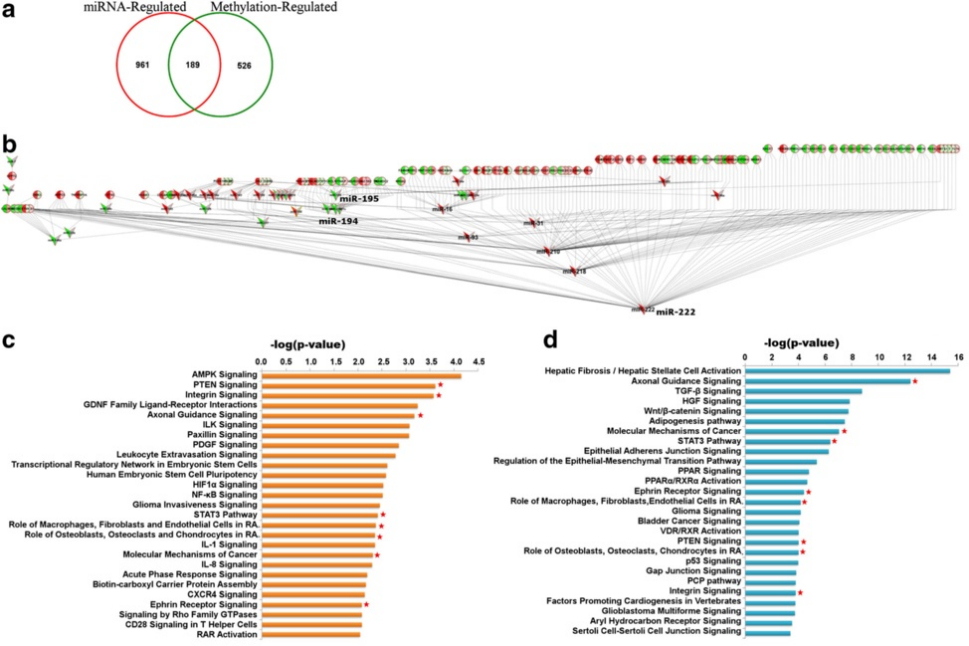
Identification of genes under miRNA and epigenetic control. **a** Venn diagram showing the overlap between differentially expressed genes regulated by differential methylation and miRNA. The analysis identified 189 genes that are under multiple regulatory controls (GMCs), which means that these genes are altered at gene expression, regulatory miRNA and methylation levels. **b** Hierarchical interaction of GMCs depicting gene expression, miRNA and epigenetic level alterations of molecules. The nodes represent the GMCs (*circles*) and the regulatory miRNA molecules (*V-shape*) and edges depict regulatory interactions. Each gene is dual colored based on expression change (log_2_FC, *left side*) and differential methylation (*right side*). *Red* denotes upregulation and *green* denotes downregulation in PDAC compared to normal. The figure also contains results from pathways enrichment analysis of (**c**) methylation-regulated genes and (**d**) miRNA-regulated genes. The y-axis represents significantly effected canonical pathways and x-axis the –log transformed Fisher’s exact test *P*-value. The common pathways are marked with *red asterisks*

The meta-analysis of transcriptome, epigenome and regulatory miRNA expression identified a large list of differentially expressed molecules associated with PDAC. Even though the results provide a significant starting point for understanding disease pathophysiology, it is difficult to pin down disease-driving molecules from long lists of genes without considering the interaction between molecules. To obtain this integral cross-talk between the differentially expressed molecules, the bimodal interactions of genes with miRNAs and genes with methylation were first interpreted. The cross-talk between differentially expressed genes and miRNAs was deduced on the basis of sequence homology-based miRNA target gene prediction. Based on predicted gene–miRNA targets, 1150 genes were identified to interact with 49 miRNAs from the PDAC meta-gene signatures and, hence, expression of these genes is potentially regulated by miRNAs (i.e., they are miRNA-regulated genes) (Fig. 4a).

The interaction between PDAC-associated gene expression and methylation changes was deciphered by comparing the differentially expressed and differentially methylated genes on the basis of HUGO gene symbols. Among the PDAC gene meta-signature, 715 genes showed overlap with the methylation meta-signature and were considered to be methylation-regulated genes (Fig. 4a).

The inverse proportionality postulate between control mechanisms and gene expression was tested by generating biplots of gene expression changes with either methylation or miRNA expression changes (Additional file 3: Figure S3). The predicted miRNA–gene interactions showed no direct or inverse correlation between gene and miRNA expression. Methylation biplots showed that hyper-methylation was associated with both upregulation and downregulation of gene expression. On the other hand, hypo-methylated genes (70 out of the 715 methylation-regulated genes) were mostly upregulated at the gene expression level (Fig. 4b). These results reinforce that the control mechanisms on gene expression are complex multi-level interactions and hence it is not possible to find a simple one-to-one relationship at a molecular level.

There were 189 genes that were common between the miRNA-regulated and methylation-regulated genes (Fig. 4a.) and thus are classified as genes under multiple regulatory controls (GMCs). Among the GMCs, the gene expression signature was ~70 % inversely correlated with the methylation signature, as expected, but the miRNA interactions did not show any significant pattern. This is probably because the miRNA–gene interactions were predicted on the basis of sequence similarity but have not been verified experimentally in pancreatic cancer. Furthermore, it has been suggested the gene–miRNA interactions do not necessarily have to be mechanisms for gene regulation, but could instead act as mechanisms for modulating cellular miRNA levels (sponge interactions) [45]. To understand this complexity and identify the functional gene–miRNA interactions in PDAC, we analyzed a PDAC dataset containing sample-matched gene and miRNA expression profiles [28, 46]. We identified the functional gene–miRNA interactions on the basis of correlation analysis of the 49 miRNAs with 1150 predicted gene targets, previously identified from the sequence similarity-based prediction. The analysis identified 36 functionally relevant miRNAs that interact with at least one of the 189 GMCs. A hierarchical network of GMCs and their functionally relevant miRNA regulators is shown in Fig. 4b. The GMC nodes are dually colored for gene expression and methylation, showing largely inverse correlation, and the miRNAs are colored based on their differential expression values.

The results from functional gene–miRNA interaction analysis show that *miR-210 *and *miR-222 *are upregulated in PDAC and are correlated with many of the GMCs (Fig. 4b). The role of *miR-210 *has been extensively studied in connection with hypoxia in the tumor micro-environment in many solid tumors, including pancreatic cancer, and its oncogenic role in cancer progression is being explored [47]. Similarly, *miR-222 *has been shown to be involved in epithelial-to-mesenchymal transition (EMT) in breast cancers, leading to an aggressive breast cancer phenotype [48]. The miRNAs *miR-194 *and *miR-195*, known to be involved in anti-tumor mechanisms in the cell, are downregulated in our PDAC signature and correlated with many of the identified GMCs in our study. For example, *miR-194 *is an epithelial marker and is known to target many genes involved in the epithelial-to-mesenchymal transition, thereby reversing the transition [49], whereas *miR-195 *has been shown to induce apoptosis in human embryonic stem cells of neural origin [50].

The comparison of methylation-regulated and miRNA-regulated genes on the basis of significantly impacted (-log(*P*-value) ≥ 10) functional categories revealed a considerable overlap between the two sets. Commonly affected functional categories include cellular growth and proliferation, cellular development, cellular movement, cell death and survival, organismal survival, organismal development, embryonic development, cancer, gastrointestinal disease, and cardiovascular system development (Additional file 4: Figure S4). Methylation-regulated genes uniquely associated with pro-inflammatory and immune response-related pathways like the *IL-1*, *IL-8* and *NFκB* signaling pathways. They were also enriched in pathways involved in cancer invasiveness and cellular motility, such as leukocyte extravasation, glioma invasiveness and Paxillin signaling pathways (Fig. 4c). The pathways specific to miRNA-regulated genes involved normal developmental pathways that regulate cell survival, cell proliferation and angiogenesis, such as *TGFβ*, *HGF*, *Wnt* and *STAT3* signaling pathways (Fig. 4d). Similar to the functional categories, we also observed a significant overlap in enriched canonical pathways between methylation-regulated and miRNA-regulated genes, including *PTEN*, integrin and axonal guidance signaling pathways (marked with asterisk in Fig. 4c, d).

**Fig. 5:**
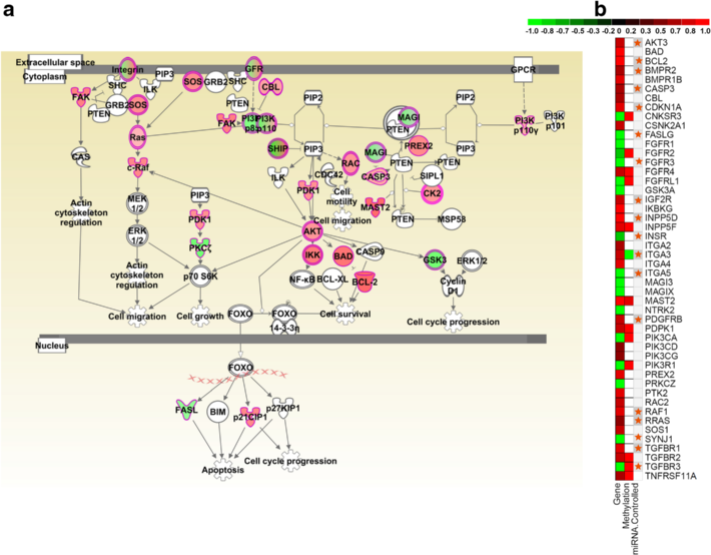
Genes under multiple regulatory controls in the *PTEN* pathway. **a** *PTEN* signaling pathway showing differentially expressed genes (*red* = upregulation, *green* = downregulation). **b** Heatmap showing differential expression (log_2_Fc) and differential methylation of dysregulated genes involved in the *PTEN* pathway. The pathway genes interacting with multiple miRNAs are marked (miR-controlled genes; *orange stars*). Dysregulated genes in the *PTEN* pathway are shown as *rows* and *columns* show differential gene expression, differential methylation and miRNA-interaction, respectively. Relative fold changes are shown with a pseudocolor scale (–1 to 1), with *red* denoting gene upregulation or hyper-methylation and *green* denoting gene downregulation or hypo-methylation

Interestingly, *PTEN* signaling, a potent pathway associated with progression and aggressiveness in multiple cancers, depicted significant dysregulation at gene expression, miRNA and methylation levels (Fig. 5a). *PTEN* is a tumor suppressor gene that functions as a phosphatase and regulates signaling pathways involved in cell growth, migration and apoptosis. *PTEN*-inactivating mutations have been associated with many cancers including prostate, breast, brain and kidney cancers. Even though *PTEN* itself is not significantly dysregulated in our PDAC meta-signatures, many genes downstream of *PTEN* are significantly dysregulated and pathways that would be inhibited by *PTEN* under normal conditions are significantly upregulated. For example, both FAK-CAS and Ras-Raf-MAPK signaling cascades involved in cell migration are upregulated, as is the *AKT* signaling pathway associated with cell survival (Fig. 5a). A heatmap of differentially expressed molecules (gene expression and methylation) involved in the *PTEN* pathway is shown in Fig. 5b, in which the miR-regulated genes are marked. Given that multiple miRNAs are known to interact and regulate these genes, individual miRNAs are not indicated in the heatmap. The methylation-based and miRNA-based regulations are occurring in different subsets of *PTEN* pathway genes, with only *ITGA3* and *TGFBR3* under multidimensional control (Fig. 5b). The hyper-methylated genes, namely *PI3K, GFR, SHIP* and *MAGI-2*, are downregulated, as would be expected, while *PDK1* and *MAST2 *are both hyper-methylated and upregulated. Even though *PI3K* is downregulated, the functions mediated by *AKT* and other downstream effectors suggest pro-survival. miRNA-mediated regulation is evidenced in the Ras-Raf-MAPK signaling cascade associated with cell migration and in pro-survival through *AKT* and *BCL2* [51].

### Systems biology analysis of the network of genes under multiple regulatory controls and identification of key regulatory hubs

One of the major shortcomings of the traditional approach is the assumption that the highly differentially expressed genes play causal roles in disease processes without differentiating driver and passenger gene expression changes. Multiple-systems biology studies have shown that the key driver genes, those playing a causal role in initiating signaling cascades that result in multiple highly differentially expressed genes, are only moderately altered in cancer and are not identified by the conventional approaches based on degree of differential expression [52]. There is also the task of narrowing down to a few critical genes from a long list of differentially expressed genes identified from these approaches. The bimodal filtering of differentially expressed genes based on alteration of upstream regulatory controls developed in this study assists in identifying a set of candidate genes with the fewest false positives and a high potential of association with disease pathophysiology. It is hypothesized that these perturbations that channel through multiple genomic spaces are the ones that perform regulatory bottle-neck roles in driving disease progression and might provide better diagnostic/prognostic or therapeutic biomarkers [53]. We took a systems-level approach to address the scale-free nature of biological systems in which a few key hubs regulate a majority of the other genes in the network [54]. The directionality of flow of perturbations can also be identified from this network-based approach and this is important in isolating the causal changes from all other cancer-associated changes.

**Fig. 6:**
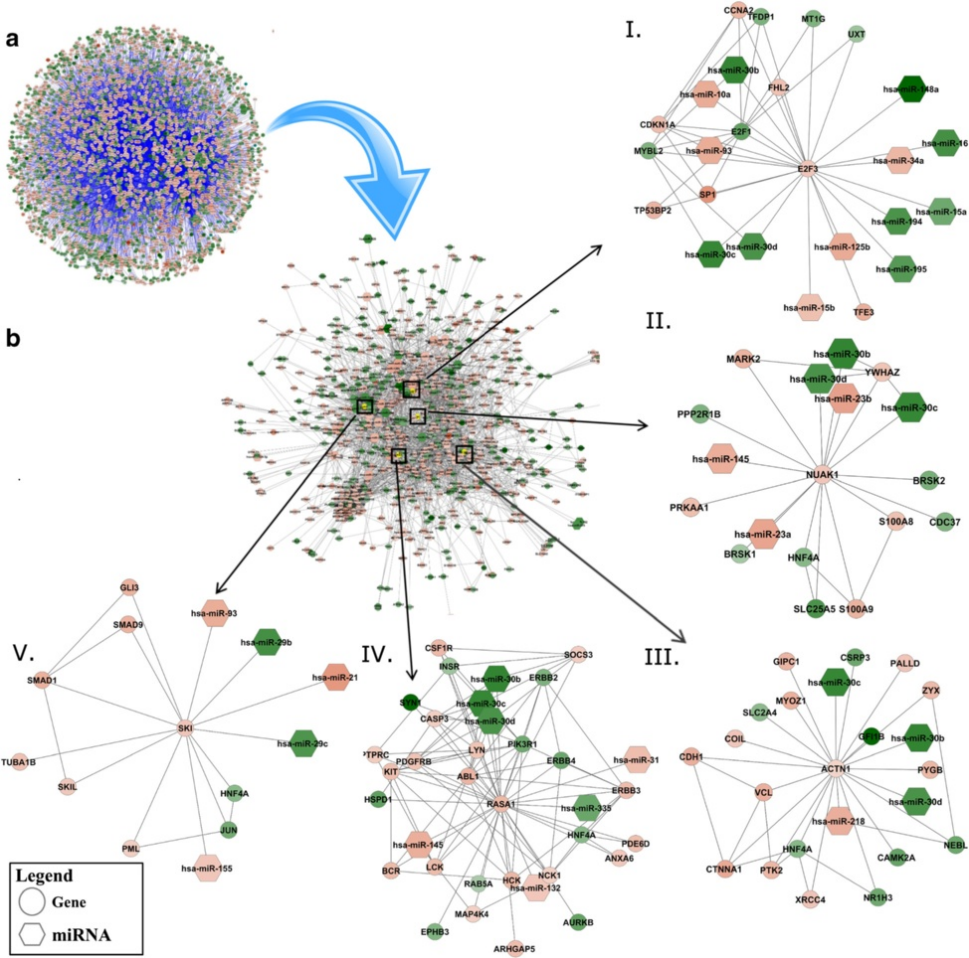
Functional gene networks of genes under multiple regulatory controls (GMCs). **a** Global network of interacting molecules obtained from multidimensional pancreatic ductal adenocarcinoma (PDAC) meta-signatures. **b** Comprehensive GMC (CGMC) network containing GMCs and their first neighbors extracted from global multidimensional PDAC network. The key regulatory hubs in the CGMC network are highlighted in *yellow*. Subnetworks (I, II, III, IV and V) of the top five key regulators (*E2F3*, *NUAK1*, *ACTN1*, *RASA1* and *SKI*) were identified from topological analysis of the CGMC network. The network genes are represented as *circles* and miRNAs as *hexagons*. Upregulated molecules are shown in shades of *red* while downregulated molecules shown in shades of *green*

The multidimensional PDAC meta-signatures were used to create a global network of 3386 nodes on the basis of available interactome information (Fig. 6a). To focus on GMCs and their interactants, a comprehensive subnetwork of 711 nodes consisting of GMCs and their first neighbors was extracted from this network (Fig. 6b). To understand the molecular processes affected by this CGMC network, we performed Fisher’s exact test-based over-representation analysis using IPA software (Qiagen). The analysis showed that the CGMC network genes were involved in many critical cellular functions related to cell cycle, growth factor signaling, apoptosis, cancer, organismal growth and proliferation, and immune response (Additional file 5: Figure S5). Among the cell cycle-related pathways, integrin signaling (*P*-value = 6.3E − 19) was the top enriched pathway with positive z-score indicating pathway activation. The *TGFβ* signaling pathway (*P*-value = 1E − 14) was the most activated and the *HGF* signaling pathway (*P*-value = 8E − 13) the most inhibited among the growth factor signaling pathways. It has been shown from analysis of TCGA RNA-seq data that *TGF-β* signaling is active in the PDAC transcriptome signature [55]. *PTEN* signaling (*P*-value = 1.9E − 14) was downregulated and was the top enriched pathway among apoptosis-related pathways. Among the cancer-related pathways, colorectal metastasis signaling (*P*-value = 1.9E − 16) and PDAC signaling (*P*-value = 1E − 13) were among the most enriched. Axonal guidance signaling and regulation of the epithelial–mesenchymal transition were the top enriched organismal growth and proliferation-related pathways. Multiple immune response-related pathways were enriched in CGMC network genes: *IL-8* and leukocyte extravasation signaling pathways were activated whereas the *CXCR4* signaling pathway was downregulated (*P*-value range = 3.9E − 15 to 3.16E − 10).

KR hubs were identified from the CGMC network through topological analysis on the basis of node interactions. The nodes were ranked based on neighborhood connectivity and node stress. From this analysis, eight KR hubs were identified (AR score ≤ 10), namely *E2F3*, *HMGA2*, *RASA1*, *IRS1*, *NUAK1*, *ACTN1*, *SKI* and *DLL1*. The top five hubs according to the AR score are shown in Fig. 6b (I–V). To understand the biological role of these KR hubs, we created KR subnetworks constituting these KR hubs along with their first neighbors and performed functional enrichment analysis of these subnetworks. *E2F3* is a top-ranking KR hub with 24 interacting neighbors that were significantly linked with G1/S transition of the mitotic cell cycle (FDR = 0.0021). The *NUAK1* subnetwork consisted of 17 neighbors associated with the *TLR* signaling pathway (FDR = 0.00041). Previous studies have shown that tumor cells express functional *TLR*s and their activation leads to tumor cell proliferation and resistance to apoptosis [56]. The *ACTN1* subnetwork had 22 interacting neighbors significantly associated with cell adhesion and cell junction organization (FDR = 8.57E − 05). The *RASA1* hub had 34 interacting neighbors involved in many growth response signaling pathways like *ERBB* signaling (FDR = 4.71E − 11). *SKI* interacted with 13 neighboring nodes in the CGMC network and was involved in endocrine system development, pancreas development and negative regulation of *TGFβ* receptor signaling (FDR = 5.07E − 05).

### Enrichment of key regulatory subnetworks in the overall comprehensive genes under multiple regulatory controls network

**Fig. 7:**
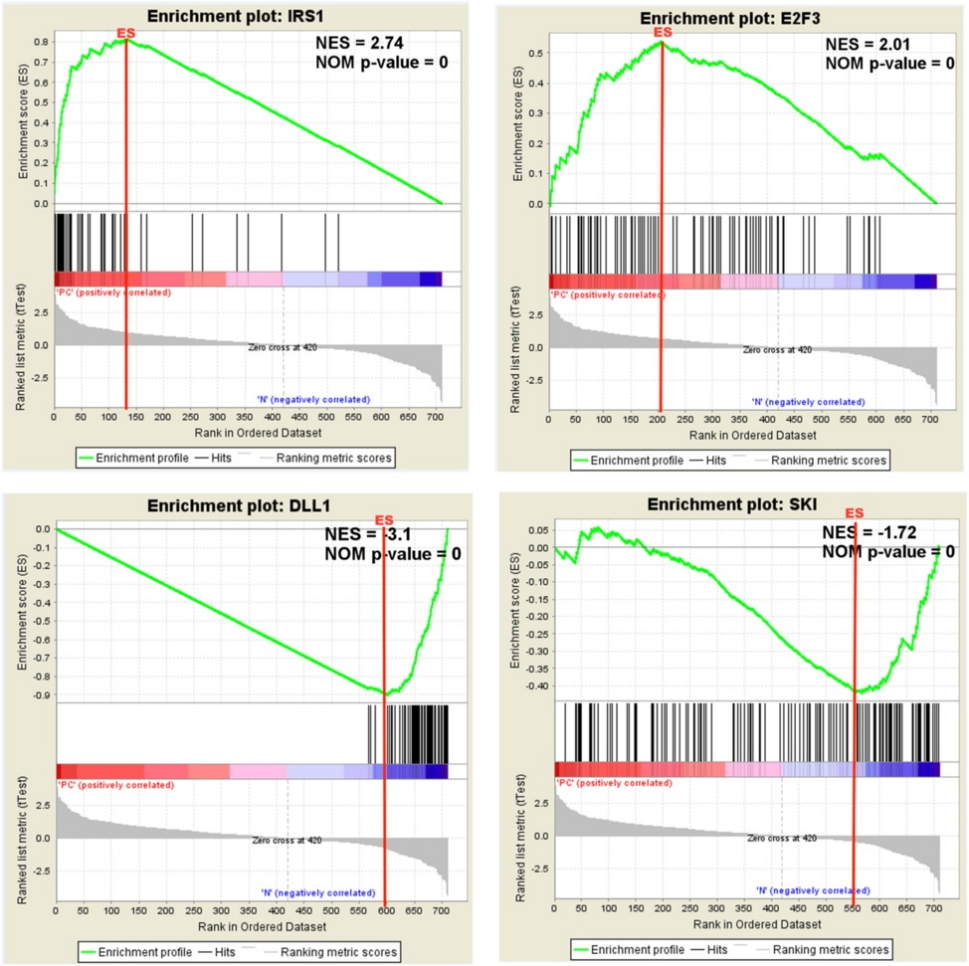
Determining the importance of key regulatory (*KR*) gene subnetworks in comprehensive genes under multiple regulatory controls (CGMC) network using gene set enrichment analysis. The figure shows enrichment plots from the top four KR subnetworks that are significantly enriched in the CGMC network. The significance was determined from nominal *P*-values (≤0.05) and false discovery rate (≤ 0.25) obtained from gene set enrichment analysis (GSEA). Each enrichment plot shows the following: Top: running enrichment score (*ES*) of the gene set (KR subnetwork) as a running-sum statistic working down the ranked list of CGMC network genes. The peak of the plot is the ES for the gene set (shown with *red line*). Middle: *black vertical bars* show the position of genes in the KR subnetwork in the ranked list of CGMC genes. The leading edge is the subset of genes that contribute most towards the ES and are the most differentially expressed genes in the CGMC network. Bottom: a *colored bar* showing positive (*red*) and negative (*blue*) correlation to phenotype and a plot showing the ranked list of CGMC genes based on the t-test that measures the gene’s correlation with the phenotype. Normalized enrichment score (*NES*) and nominal *P*-value (*NOM*) obtained from the GSEA analysis are shown on top of each plot

The importance of the identified KRs in the CGMC network was assessed using a GSEA approach. The analysis was designed to understand the importance of subnetworks created from eight identified KRs in the overall CGMC network. The KRs were used as seeds to partition the CGMC network into PAM clusters using the k-medoids clustering methodology. This methodology identifies subnetworks on the basis of correlation of KRs with other genes in the network instead of just taking into consideration the first neighbors of KRs. The enrichment of these KR clusters in the CGMC network was assessed using GSEA. Six of the eight KR clusters showed statistically significant enrichment (nominal *P*-value ≤ 0.05 and FDR ≤ 0.25) (Table 2), confirming that these KRs and their correlated genes (KR subnetworks) comprise the most altered genes in PDAC among the CGMC genes. Figure 7 shows the GSEA enrichment plots of the top four of these KR clusters, namely *IRS1*, *E2F3*, *DLL1* and *SKI*. The *IRS1* cluster had the highest positive enrichment score (normalized enrichment score = 2.74) and the *DLL1* cluster had the highest negative enrichment score (normalized enrichment score = −3.1). *ACTN1* and *HMGA2* clusters showed a general overall distribution of genes in the network skewed more towards the upregulated genes. *SKI* showed a similar trend but skewed more towards the downregulated genes in the network. The results clearly show that even though we started with a set of differentially expressed genes that were not filtered based on fold change, the KRs and their subnetworks are formed from substantially differentially expressed genes. This subnetwork enrichment analysis has provided a way to understand the relative importance of the identified KRs in stabilizing PDAC network. The analysis also showed that *IRS1*, *E2F3*, *DLL1*, *SKI*, *ACTN1* and *HMGA2* are correlated with genes that are more differentially expressed in PDAC compared to *RASA1* and *NUAK1*.

**Table 2:** Gene set enrichment analysis of KR-PAM clusters

Name	Size	ES^a^	NES^b^	Nom *P*-value^c^	FDR q-value^d^
IRS1	47	0.814297	2.749994	0	0
E2F3	83	0.538403	2.012985	0	0
ACTN1	197	0.364171	1.523528	0	0.018332
HMGA2	75	0.401607	1.467728	0.0122378	0.020817
NUAK1	26	0.332612	1.002291	0.4466912	0.4542
DLL1	57	−0.90119	−3.20045	0	0
SKI	133	−0.42133	−1.72121	0	0.001876
RASA1	93	−0.19612	−0.75914	0.9367682	0.91951

^a^ *ES* Enrichment score showing the maximum deviation from zero based on Kolmogorov-Smirnov statistic

^b^ *NES* Normalized enrichment score calculated from a null distribution created from 1000 permutations of phenotype labels

^c^ Nominal *P*-value obtained from the null distribution

^d^ Multiple test corrected *P*-value

*FDR* false discovery rate

### Survival analysis of identified key regulators in pancreatic cancer

The significance of the KRs was further tested using survival analysis on the eight identified KRs in two PDAC expression datasets. The samples were partitioned into two groups by the most contrasting expression characteristics for the eight KRs using the SOM clustering approach, and survival analysis was performed on the two clusters (Additional file 6: Figure S6; Ai. and Bi). The results showed that the KRs were able to clearly discriminate between better versus poor survivors (*P*-values 0.0368–0.0464), indicating their prognostic role in PDAC.

**Fig. 8:**
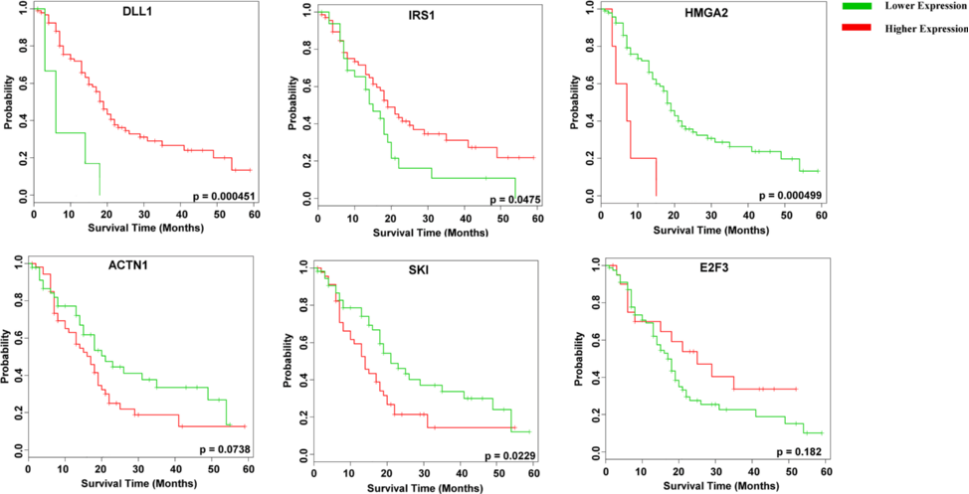
Survival association analysis of individual key regulators (*KRs*) in pancreatic ductal adenocarcinoma survival plots of KRs, depicting significant correlation (log-rank test *P*-value ≤ 0.075) between KR expression and patient survival time. Lower expression levels of *HMGA2*, *ACTN1*and *SKI *and higher expression levels of *IRS1* and *DLL1* were associated with better survival probabilities. The log-rank *P*-value between the two groups created based on the mean or median split of the expression values of KRs is shown at the bottom of each plot

To further ascertain the relative importance of these eigth identified KRs in survival, we performed survival analysis on individual KR genes. In the individual gene-based survival analysis, *HMGA2*, *IRS1*, *ACTN1*, *SKI* and *DLL1* showed significant differences in survival probabilities (log-rank test *P*-value ≤ 0.075) when samples were split on the basis of low or high expression of individual KRs (Fig. 8). The analysis showed that higher expression levels of *IRS1* and *DLL1* and lower expression levels of *HMGA2*, *ACTN1* and *SKI* were associated with significantly higher survival probabilities. Interestingly, these five KR genes that show higher prognostic value in PDAC among the KRs also comprise the most enriched subnetworks in the GSEA analysis (five out of the six enriched KR subnetworks). *E2F3*, which was the other KR that was significant in the GSEA-based subnetwork enrichment analysis, did not reach statistical significance in the individual survival analysis, but was able to discriminate poor survivors from better survivors to some extent. Thus 83 % of the KRs filtered through subnetwork enrichment analysis were also found to have significant prognostic value in the survival analysis, indicating that our systematic filtering approach has provided a valuable set of genes that are potential biomarkers with prognostic value in PDAC.

## Discussion

One of the most challenging tasks in the analysis of large-scale *omics* data is the identification of the causal (KR) changes and differentiating them from bystander effects associated with the disease. Owing to the large number of genes measured in genomic assays, it is difficult to achieve statistical power except for the most highly differentially expressed molecules. In many cases the KRs remain unidentified owing to subtle changes at expression level. Even when identified as being differentially expressed, KRs are hard to characterize based on just their differential expression measure from a long list of altered molecules. However, the alternative approach of identifying regulatory genes from biochemical studies is laborious and time consuming and does not compare to genome-scale experiments in providing comprehensive coverage.

Numerous studies have shown that network-based approaches that take into consideration the contribution of individual genes along with their interactions are an effective solution in identifying KRs of disease pathophysiology [57]. It has been hypothesized that KRs are functional bottlenecks in disease initiation and progression because they regulate the expression of a large number of downstream effector genes [57]. Most of these studies performed the network analysis on gene expression data, ignoring the contribution of upstream regulatory genomics and epigenetics spaces. We hypothesize that molecules whose alterations result in upstream regulatory changes in a feedback manner as well as in important downstream effector changes are critical for disease progression and pathophysiology.

Keeping this in mind, we adopted a multistep approach integrating the cross-talk between different genomics spaces (the epigenome, transcriptome and regulatory miRNAs) as well as an interactive network analysis to identify the key driver genes associated with PDAC. To achieve this, we initially performed rank-based meta-analysis individually on epigenetic, transcriptional and miRNA expression data to define a comprehensive landscape of molecular alterations in PDAC. It is well established that rank-based methods perform more reliably and consistently than non-parametric and parametric methods for meta-analysis of microarray data [24]. In this study we implemented a rank-based meta-analysis approach, which enabled us to identify consistently differentially expressed molecules from multiple studies and increased the statistical power by eliminating false-positive genes from individual studies while retaining consistently differentially expressed molecules with subtle changes.

To generate a comprehensive view of alterations associated with PDAC as well as understanding the cross-talk among different genomic spaces, we adopted a multidimensional modeling-based approach. This step involved the integration of meta-analysis results from each *omics* dimension (the epigenome, transcriptome and regulatory miRNAs) and is important for understanding the regulatory cascades and cross-talk between gene expression and upstream regulatory mechanisms. Understanding of the role of these epigenetic and post-transcriptional modifications in the development of disease phenotype could be crucial to identify regulatory bottlenecks that play causal roles in the disease. Profound epigenetic changes have been shown to associate with the onset and progression of cancer, and changes in DNA methylation is one of the potent underlying mechanisms [58, 59]. Similarly, significant dysregulation has been reported in regulatory miRNA expression in cancer [60, 61]. In fact, it has been shown that *miR-21* and *miR-222*, which formed part of our multidimensional PDAC meta-signature, are both probable prognostic markers for PDAC [61]. It is important to note that *miR-222 *was also identified as a KR of PDAC pathophysiology in our systems-level network analysis. In this respect, the interactive network analysis of miRNA subnetworks created from the global PDAC meta-signatures network reiterated the importance of the systems-level approach in understanding the cause–effect relationship between the dysregulated molecules (Additional file 7: Figure S7). The analysis showed that *miRNA-221 *and *miRNA-222 *were highly correlated and were at the center of a subnetwork created around *miR-222* (Additional file 7: Figure S7B), while the highly dysregulated *miRNA-210 *was one of the peripheral molecules in its subnetwork (Additional file 7: Figure S7A). In a conventional analysis of dysregulated genes, all three miRNAs would have been weighted equally in their contribution to disease pathophysiology, whereas a systems-level analysis clearly shows that *miRNA-221 *and *miRNA-222 *play a more causal role than *miRNA-210 *in PDAC disease progression. It was also evident from our analysis that *miR-194*, an epithelial marker that is downregulated in PDAC, is the center of the subnetwork created from *miR-210*.

It has been postulated that dysregulation in regulatory mechanisms can play important causal roles in disease initiation and/or progression [61, 62]. In many instances, these regulatory layers provide the missing links to rationalize the observed variance seen in gene expression. It is also established that regulatory miRNAs are themselves regulated by epigenetic changes and sponge miRNA targets [45], which would be another layer of regulatory control that needs to be factored into our multidimensional model in the future. We overcame this additional complexity here by including only the miRNA changes that were correlated with gene expression changes from *multi-omics* studies with both gene expression and miRNA expression data on matched samples. Overall, in the multidimensional modeling step we identified 189 genes that manifested concerted changes across gene expression and their regulatory mechanisms in pancreatic cancer. As expected, this consensus multidimensional PDAC signature was enriched in important pathways associated with cancer progression, such as cell cycle regulation, cell growth and proliferation, cell motility, cell-based immunity, and inflammatory response.

Further, to understand the cause–effect relationship and the mechanistic overview of PDAC progression, we performed interaction network analysis on GMCs on the basis of interactome information. This multidimensional network was analyzed for topological cues to identify the most probable candidates for KRs of the network and, by extension, key disease modulators. These KR molecules represent regulatory bottlenecks and play a causal role in disease initiation and progression. The presence of these regulatory bottlenecks in establishing disease phenotype has been shown in multiple studies relating to cancer [57, 63–68]. These studies showed that genes with no prior known oncogenic status or known mutations associated with a particular disease could still be influential in controlling many disease-related genes, and thus serve as regulatory bottlenecks that can be identified only by a systems-level approach. In our analysis, ranking based on a node centrality measure (node stress) and neighborhood connectivity identified eight probable regulatory hubs in the network. Thus, our consensus multidimensional approach along with the systems-level network analysis resulted in a dramatic reduction of testable hypotheses from 6863 molecules to eight genes. Finally, the ability to identify the KRs of disease processes independent of sample-matched *omics* profiles would be a great advantage that allows, in theory, the use of all available genomics data associated with the disease. However, this also imposes a huge responsibility of maintaining consistency in the subtype context across the multiple dimensions of analysis to produce meaningful and concordant results.

## Conclusions

In this study we were able to isolate converging regulatory modules and KR molecules associated with PDAC pathophysiology by employing a hierarchical systems-level multidimensional data analysis approach. GSEA on subnetworks of these KRs depicted significant enrichment in the leading edges of the CGMC network genes, suggestive of their important roles in PDAC progression. The identified KRs were able to differentiate poor survivors from better survivors, further strengthening our evidence that they are genes of prognostic value and can be used as probable therapeutic targets in treatment of pancreatic cancer. Data integration methods for multidimensional data are still in their infancy, but *multi-omics* integration using new experimental and computational approaches is already producing useful functional models and meaningful insights into disease pathophysiology.

## Additional files

Additional file 1: Figure S1. Method schematic. Schematic representation of methodology used in the study. The major steps are shown in gray, the methods or applications used in different steps are indicated on the arrows leading to the outcomes of each step shown in blue/pink. Final outcome is shown in orange. (PDF 313 kb) Additional file 2: Figure S2. Conventional differential expression analysis of transcriptome and regulatory miRNA compared to the RP method. The results from traditional limma analysis on individual datasets are shown at the top and results from the individual RP analysis at the bottom of the figure. **A** Venn diagram showing the overlap of differentially expressed genes obtained from multiple mRNA datasets (upregulated genes shown on the left and downregulated genes on the right). **B** Venn diagram showing the overlap of differentially expressed miRNAs obtained from multiple miRNA datasets. RP analysis identified more commonly differentially expressed genes compared to limma, therefore RP was chosen for meta-analysis. (TIFF 21797 kb) Additional file 3: Figure S3. Biplots of dysregulated genes with interacting dysregulated miRNAs and differential methylation. **A** Biplots showing logFc of genes along x-axis and logFc of interacting miRNAs along y-axis. **B** Biplots showing logFc of genes (x-axis) and logFc of differential methylation for the same genes (y-axis). Upregulated and down regulated genes are denoted in red and green, respectively. A gene–miRNA biplot showed no discernible distribution pattern while gene–methylation biplots showed that hypomethylated genes are mostly upregulated. (TIFF 4729 kb) Additional file 4: Figure S4. Functional enrichment analysis of miRNA-regulated and methylation-regulated genes. Functions associated with (**A**) miRNA-regulated genes and (**B**) methylation-regulated genes. The y-axis represent significantly effected functions and the x-axis represents log-transformed Fisher’s exact test *P*-value. (TIFF 16066 kb) Additional file 5: Figure S5. Pathways enrichment analysis of CGMC network genes. Pathways associated with CGMC network genes with a − log-transformed multiple test-corrected Fisher’s exact test *P*-value (NLP) ≥10 are shown. Bar graphs showing enriched pathways (x-axis). Pathways are sorted on the basis of multiple test corrected NLP (y-axis). The bars are colored based on enrichment score (z-score); positive z-scores shown in orange indicate probable activation of the pathway, negative z-scores shown in blue indicate probable suppression of the pathway. (TIFF 10783 kb) Additional file 6: Figure S6. Survival association analysis of KRs in PDAC. **A** Results from PDAC survival dataset 1 showing (i) partitioning of samples on the basis of expression profile of KRs using SOM clustering method. We identified two strikingly opposite expression patterns (black and red ellipses). (ii) Kaplan–Meier plots showing survival association of clusters 1 (black) and 2 (red) that correspond to the opposing expression patterns from i. Log-rank test *P*-value shown on top of the survival plot. (iii) Heatmap showing expression of KR genes in clusters 1 and 2. The column side bars show cluster 1 (black) and cluster 2 (red). **B** Results from similar analysis of PDAC survival dataset 2. In the heatmap, samples are shown as columns and KR genes as rows. Relative expression shown with a pseudocolor scale (−1 to 1), with red denoting relative high expression and green denoting relative low expression in row-scaled data. (TIFF 35079 kb) Additional file 7: Figure S7. miRNA-centered interactive networks obtained from dysregulated functionally relevant miRNAs in PDAC. **A** Network built around *miR-210 *from global multidimensional PDAC signatures network. The visual analysis of the network indicates *miR-194 *as a KR instead of *miR-210 *(thick gray outline). **B** Networks built similarly around *miR-221*. It is centered on *miR-221 *and *miR-222 *(thick black outline). Genes represented as circles and miRNAs as hexagons; first neighbors of the *miR-210 *and *miR-221 *highlighted in thick blue outline. (TIFF 26867 kb)
