# Supplementary figures and images for: Identification of key regulators of pancreatic cancer progression through multidimensional systems-level analysis
Source: Genome Med. 2016 May 3;8:38. doi: 10.1186/s13073-016-0282-3 (PMC4853852; doi:10.1186/s13073-016-0282-3)

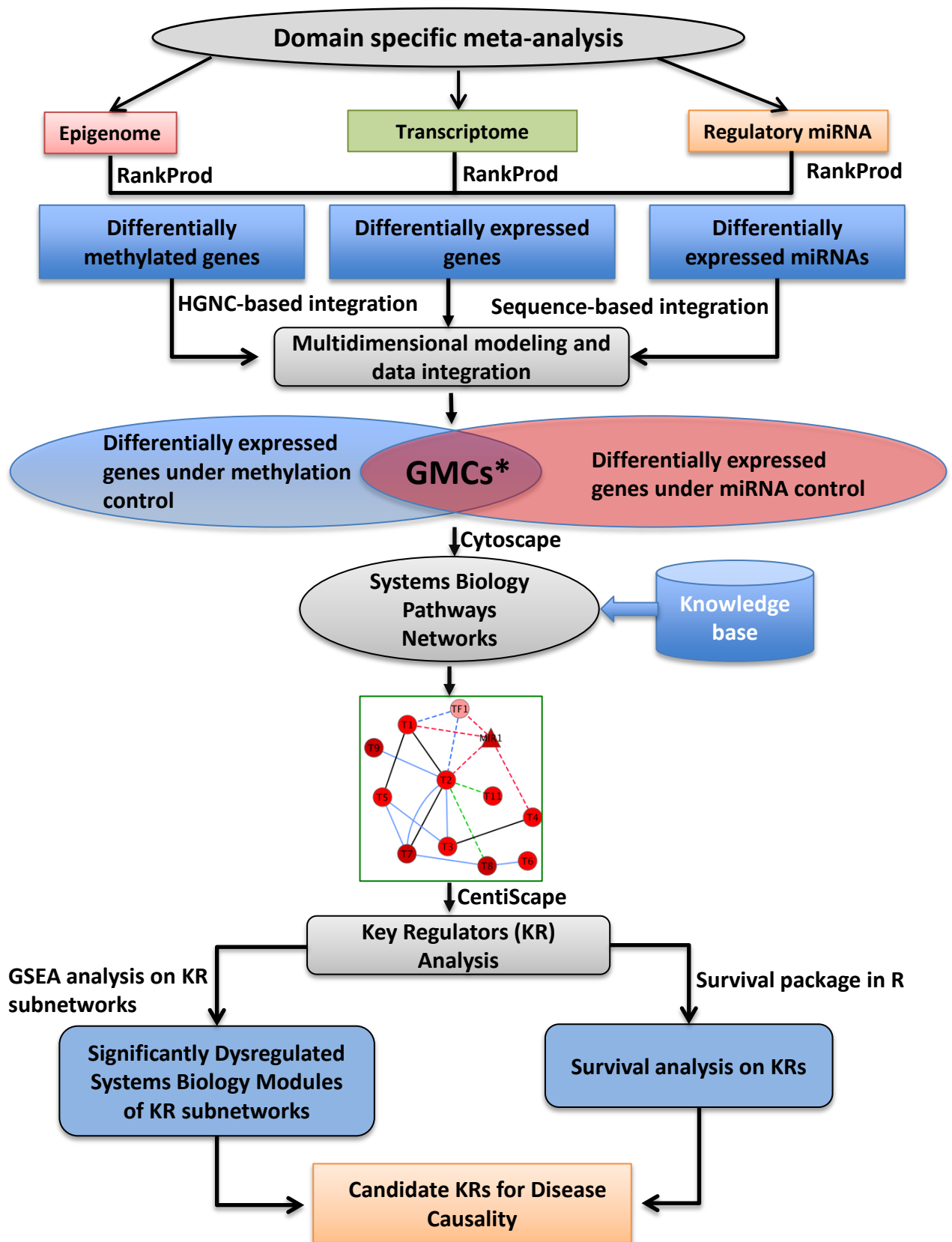

\* Genes under Multiple regulatory Control

Supplement: Additional file 1: Figure S1. — Method schematic. Schematic representation of methodology used in the study. The major steps are shown in gray, the methods or applications used in different steps are indicated on the arrows leading to the outcomes of each step shown in blue/pink. Final outcome is shown in orange. (PDF 313 kb) [file 13073_2016_282_MOESM1_ESM.pdf]

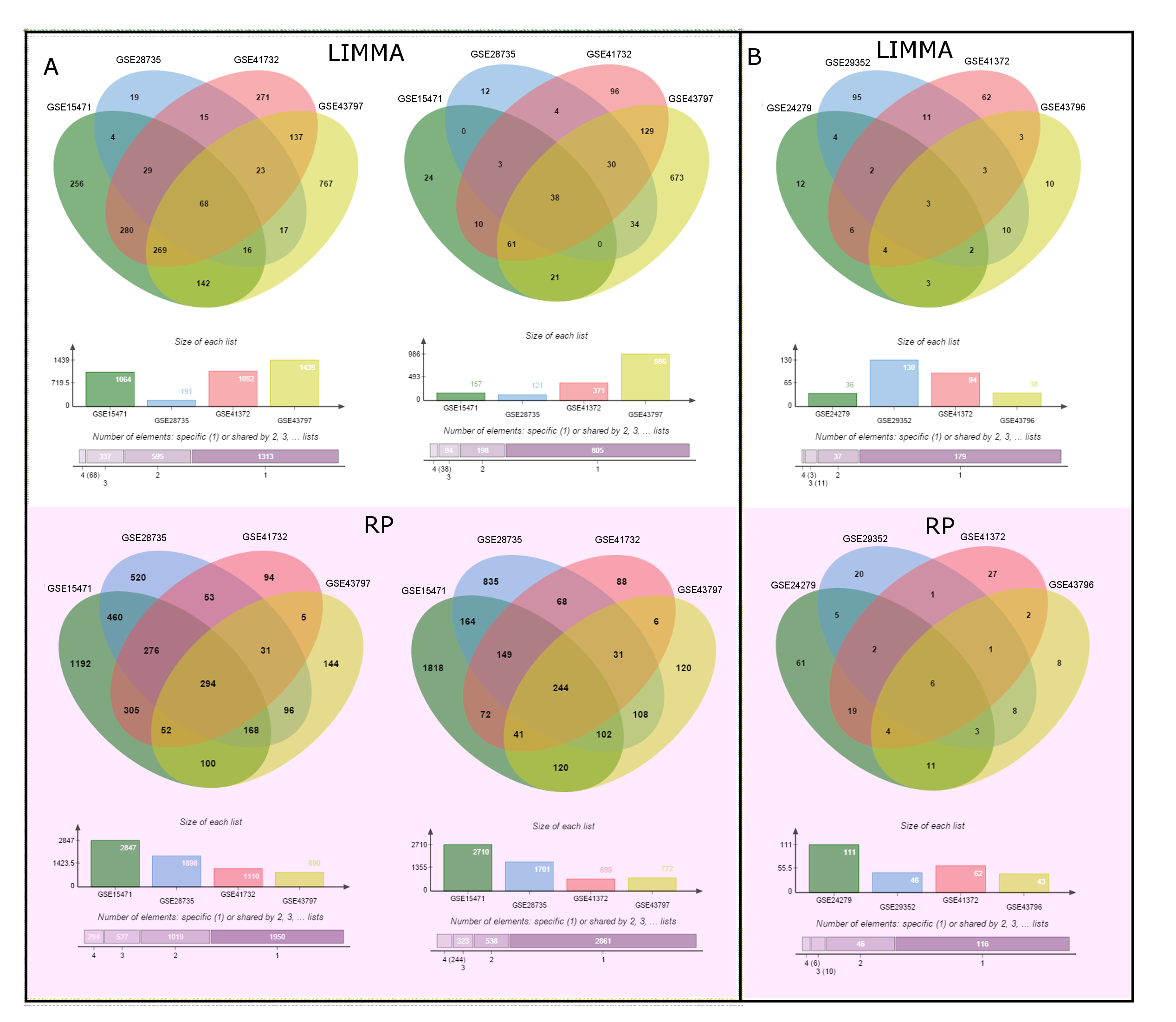

Supplement: Additional file 2: Figure S2. — Conventional differential expression analysis of transcriptome and regulatory miRNA compared to the RP method. The results from traditional limma analysis on individual datasets are shown at the top and results from the individual RP analysis at the bottom of the figure. A Venn diagram showing the overlap of differentially expressed genes obtained from multiple mRNA datasets (upregulated genes shown on the left and downregulated genes on the right). B Venn diagram showing the overlap of differentially expressed miRNAs obtained from multiple miRNA datasets. RP analysis identified more commonly differentially expressed genes compared to limma, therefore RP was chosen for meta-analysis. (TIFF 21797 kb) [file 13073_2016_282_MOESM2_ESM.tiff]

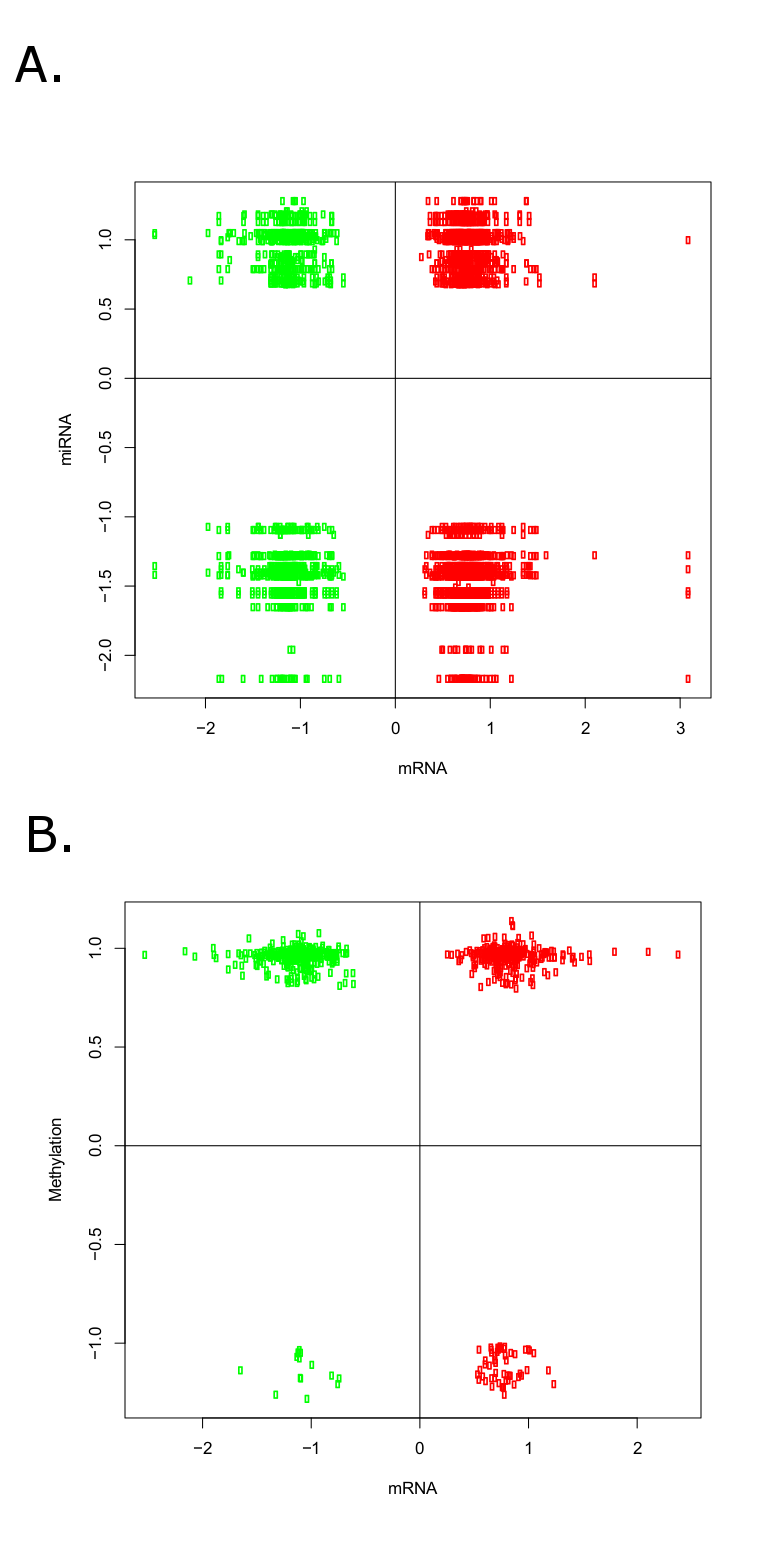

Supplement: Additional file 3: Figure S3. — Biplots of dysregulated genes with interacting dysregulated miRNAs and differential methylation. A Biplots showing logFc of genes along x-axis and logFc of interacting miRNAs along y-axis. B Biplots showing logFc of genes (x-axis) and logFc of differential methylation for the same genes (y-axis). Upregulated and down regulated genes are denoted in red and green, respectively. A gene–miRNA biplot showed no discernible distribution pattern while gene–methylation biplots showed that hypomethylated genes are mostly upregulated. (TIFF 4729 kb) [file 13073_2016_282_MOESM3_ESM.tiff]

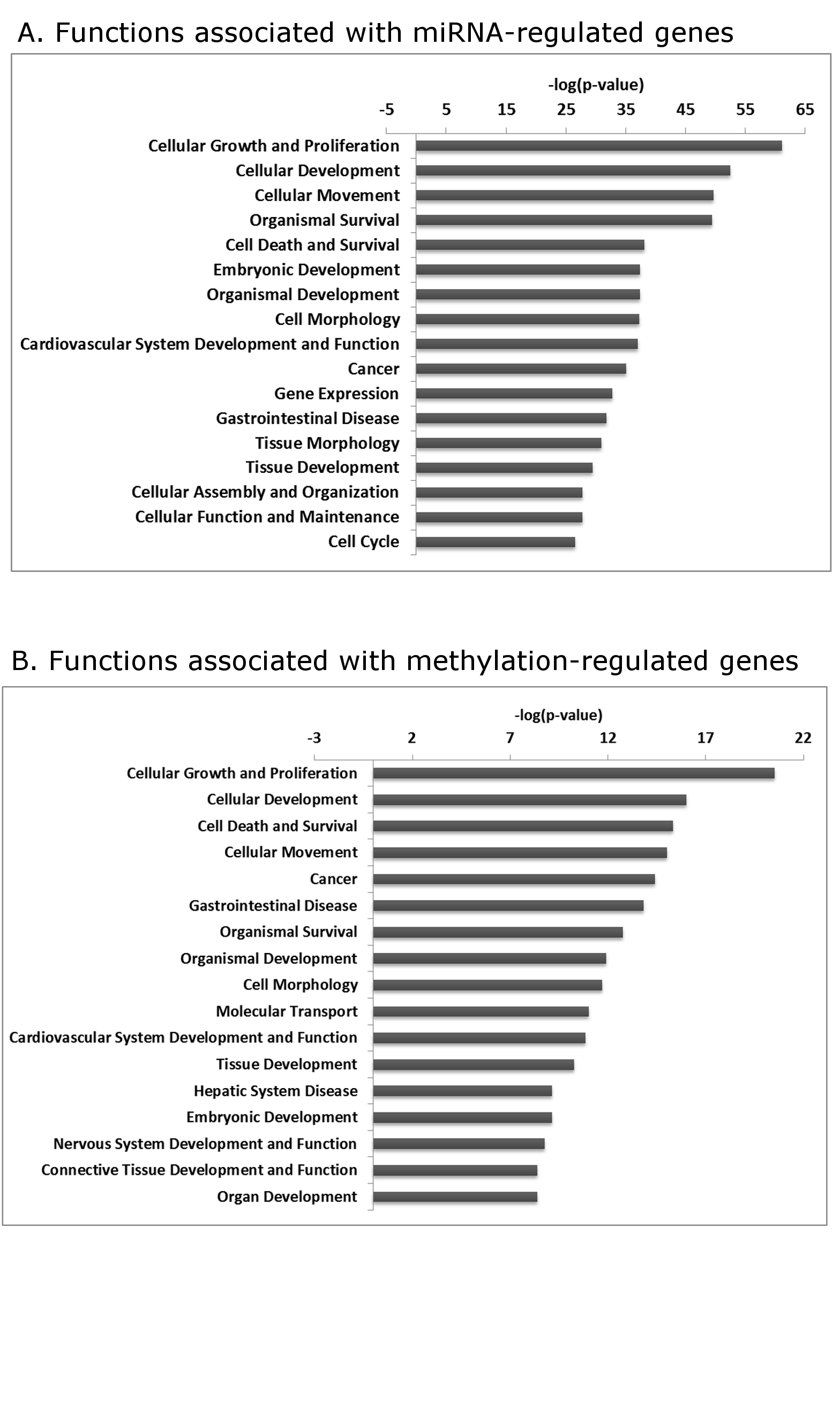

Supplement: Additional file 4: Figure S4. — Functional enrichment analysis of miRNA-regulated and methylation-regulated genes. Functions associated with (A) miRNA-regulated genes and (B) methylation-regulated genes. The y-axis represent significantly effected functions and the x-axis represents log-transformed Fisher’s exact test P-value. (TIFF 16066 kb) [file 13073_2016_282_MOESM4_ESM.tiff]

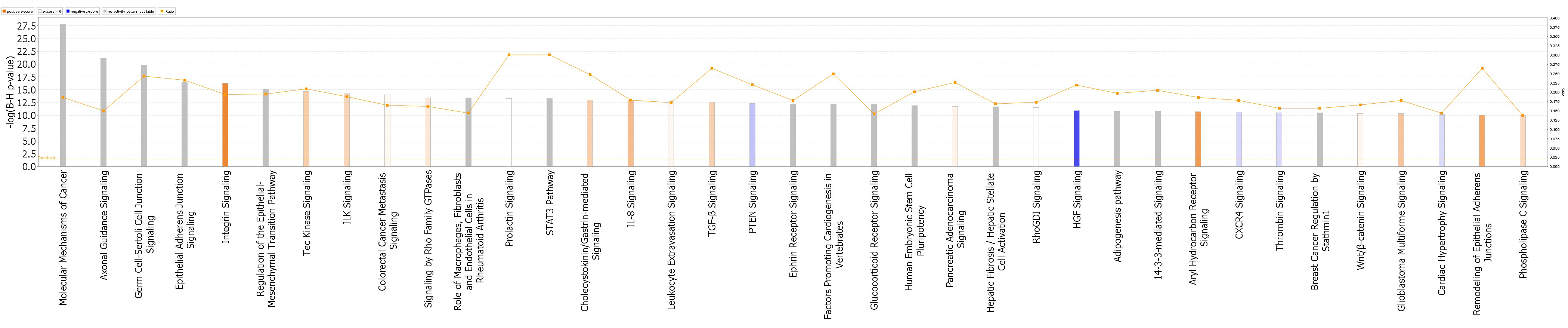

Supplement: Additional file 5: Figure S5. — Pathways enrichment analysis of CGMC network genes. Pathways associated with CGMC network genes with a − log-transformed multiple test-corrected Fisher’s exact test P-value (NLP) ≥10 are shown. Bar graphs showing enriched pathways (x-axis). Pathways are sorted on the basis of multiple test corrected NLP (y-axis). The bars are colored based on enrichment score (z-score); positive z-scores shown in orange indicate probable activation of the pathway, negative z-scores shown in blue indicate probable suppression of the pathway. (TIFF 10783 kb) [file 13073_2016_282_MOESM5_ESM.tiff]

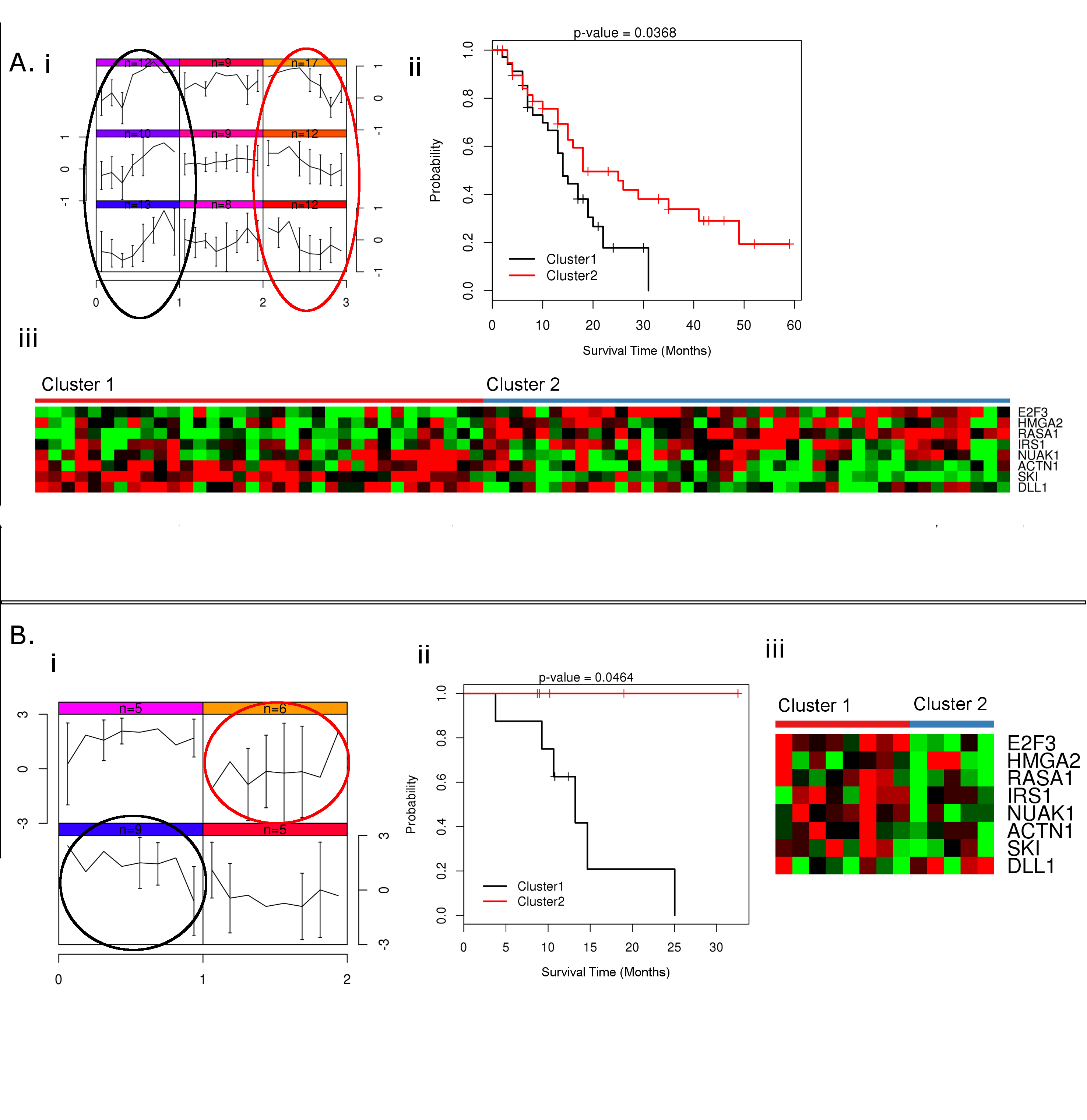

Supplement: Additional file 6: Figure S6. — Survival association analysis of KRs in PDAC. A Results from PDAC survival dataset 1 showing (i) partitioning of samples on the basis of expression profile of KRs using SOM clustering method. We identified two strikingly opposite expression patterns (black and red ellipses). (ii) Kaplan–Meier plots showing survival association of clusters 1 (black) and 2 (red) that correspond to the opposing expression patterns from i. Log-rank test P-value shown on top of the survival plot. (iii) Heatmap showing expression of KR genes in clusters 1 and 2. The column side bars show cluster 1 (black) and cluster 2 (red). B Results from similar analysis of PDAC survival dataset 2. In the heatmap, samples are shown as columns and KR genes as rows. Relative expression shown with a pseudocolor scale (−1 to 1), with red denoting relative high expression and green denoting relative low expression in row-scaled data. (TIFF 35079 kb) [file 13073_2016_282_MOESM6_ESM.tiff]

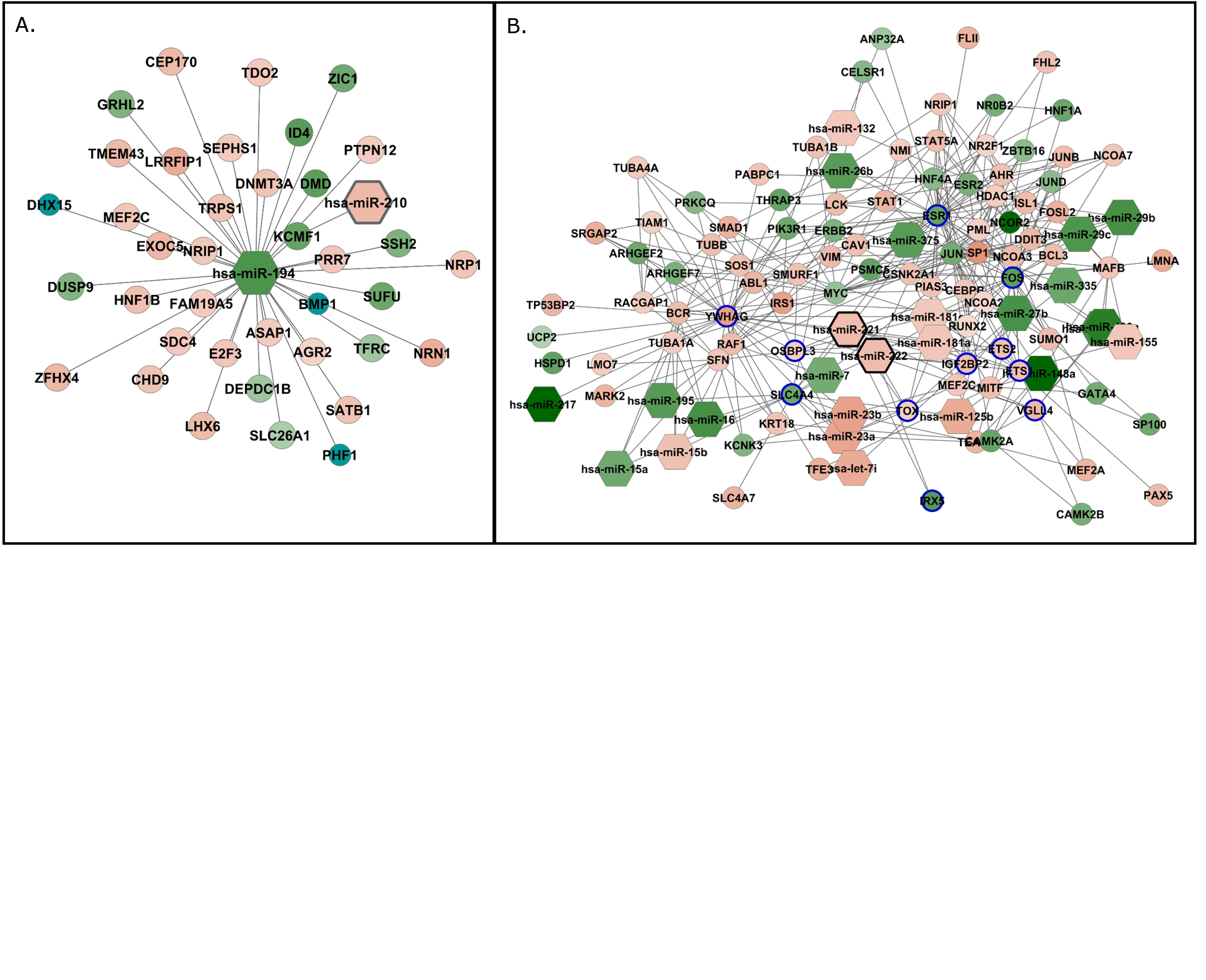

Supplement: Additional file 7: Figure S7. — miRNA-centered interactive networks obtained from dysregulated functionally relevant miRNAs in PDAC. A Network built around miR-210 from global multidimensional PDAC signatures network. The visual analysis of the network indicates miR-194 as a KR instead of miR-210 (thick gray outline). B Networks built similarly around miR-221. It is centered on miR-221 and miR-222 (thick black outline). Genes represented as circles and miRNAs as hexagons; first neighbors of the miR-210 and miR-221 highlighted in thick blue outline. (TIFF 26867 kb) [file 13073_2016_282_MOESM7_ESM.tiff]
